# Physical Activity in Malaysia: Are We Doing Enough? Findings from the REDISCOVER Study

**DOI:** 10.3390/ijerph192416888

**Published:** 2022-12-15

**Authors:** Nik Munirah Nik-Nasir, Mazapuspavina Md-Yasin, Farnaza Ariffin, Nafiza Mat-Nasir, Maizatullifah Miskan, Najmin Abu-Bakar, Khalid Yusoff

**Affiliations:** 1 Primary Care Medicine Department, Faculty of Medicine, Universiti Teknologi MARA, Sungai Buloh 47000, Malaysia; 2 Primary Care Medicine Unit, Faculty of Medicine and Defence Health, National Defence University of Malaysia, Kuala Lumpur 57000, Malaysia; 3 Centre for Translational Research and Epidemiology (CenTRE), Faculty of Medicine, Universiti Teknologi MARA, Sungai Buloh 47000, Malaysia

**Keywords:** physical activity, prevalence, patterns of physical activity, IPAQ, Malaysia

## Abstract

Physical activity (PA) in the form of structured or unstructured exercise is beneficial for health. This paper aims to study PA levels across four domains according to the International Physical Activity Questionnaire (IPAQ) and its associated factors. A total of 7479 Malaysian adult participants between 18 to 90 years old from the REDISCOVER study who completed the IPAQ were analyzed. PA was calculated as MET-min per week and were categorized according to insufficiently active, sufficiently active and very active. Multinomial regression was used to determine the association between sociodemographic, clinical factors and the level of PA. The mean age of the participants was 51.68 (±9.5 SD). The total reported physical activity in median (IQR) was 1584.0 (0–5637.3) MET-min per week. The highest total for PA was in the domestic domain which is 490 (0–2400) MET-min per week. Factors associated with sufficiently active or very active PA include Malay ethnicity, no formal education, elementary occupation, current smokers and high HDL. Whereas low income, male and normal BMI are less likely to participate in sufficiently active or very active PA. Intervention to encourage higher PA levels in all domains is important to achieve recommended PA targets.

## 1. Introduction

The benefits of physical activity have been well established, ranging from risk reduction for premature cardiovascular death to development of non-communicable diseases such as ischemic heart disease, stroke, type 2 diabetes and cancer [1,2,3]. According to the World Health Organization (WHO), physical activity is defined by any bodily movement produced by skeletal muscles that requires energy expenditure [4]. In general, physical activity can be divided into structured or unstructured exercise programs and non-exercise physical activity such as standing, walking, performing house chores and occupational-related activities. Based on the International Physical Activity Questionnaire (IPAQ), these types of physical activity can be categorized into work, active transportation, domestic and yard work, and leisure time domains [5].

The positive outcomes from physical activity are not only limited to the standard recommendation of 150 min per week of moderate to vigorous physical activity (MVPA). Previous research has shown that any form of physical activity is desirable compared to none at all [6,7]. This is supported by a systematic review of cohort studies, which found that the risk ratio in terms of protective effect against all-cause mortality for total physical activity, which include occupation, daily living and leisure [(0.65 (95% CI 0.60–0.71)] and physical activities of daily living only [0.64; 95% CI 0.55–0.75], is at par with exercise and sports [0.66; 95% CI 0.61–0.71] (CI = confidence interval) [8].

Despite various health programs and campaigns, the prevalence of the population achieving the standard recommendation of 150 min per week of MVPA remained modest [9,10,11]. The current physical inactivity prevalence continues to increase in high income countries compared to low-income countries [12]. The Global Action Plan on Physical Activity 2018–2030 calls for overall improvement of physical activity based on the 2016 baseline prevalence among adults and adolescents by targeting 15% reduction in physical inactivity [13].

Malaysia is a Southeast Asian country boasting a population of 32.7 million in the year 2022. Its population consists of multiracial ethnicities with 75.1% of its people living in cities [14]. As Malaysia continues to top the charts for the prevalence of obesity in the region of Southeast Asia [15], the slow incline in physical activity rates requires an urgent plan of action [16]. Data on physical activity patterns and its correlates are vital in guiding multi-agency intervention programs to improve physical activity levels in the population. Previous literature has reported the physical activity patterns and factors associated with physical activity [17,18,19,20,21]. Evidence from the National Health and Morbidity Survey (NHMS) 2019 showed that the prevalence of physical inactivity among adults in Malaysia was 24.6%; which is higher in comparison to other Asian countries such as China, India and Hong Kong [22]. This brings us to the subject of interest, which is to explore the patterns of physical activity among the Malaysian population based on the four IPAQ domains, which are work, active transportation, domestic and yard work, and leisure time and factors associated with physical activity levels.

## 2. Materials and Methods

### 2.1. Study Design

The REDISCOVER (Responding to Increasing Cardiovascular Disease Prevalence) study is a large-scale ongoing prospective community-based cohort study involving 13,654 Malaysian adults in urban and rural communities. The study began in 2007, and data collection is ongoing. The study duration is 25 years, and data collection is repeated every three years. The methodology for the REDISCOVER Study has been published elsewhere [23,24,25,26], whereby specific methods were highlighted as relevant to the sub topics. In previous publications, the number of baseline data was different, as the baseline data collection was still ongoing.

### 2.2. Site Selection

The study site involved 18 urban and 22 rural communities. The states involved were Selangor, Negeri Sembilan, Pahang, Kelantan, Sarawak and Sabah, and the Federal Territory of Kuala Lumpur. The five states were pragmatically selected to ensure sufficient representation of the major ethnic groups in Malaysia. The main ethnic groups in Peninsular Malaysia are Malays, Chinese and Indian, while the main ethnic groups in Sabah are the Kadazan-Dusun, Bajau and Murut. In Sarawak, the main ethnic groups are Melanau and Iban. For the purpose of this study, the Kadazan-Dusun, Bajau, Murut, Melanau, Iban and several other ethnic minorities from Sabah and Sarawak represent the indigenous population of Bumiputera. States such as Selangor, Negeri Sembilan and Pahang were chosen in view of having a good mixture of Malay, Chinese and Indian populations. Kelantan, on the other hand, offers a predominantly Malay population.

Urban and rural areas were defined according to the Malaysian Population and Housing Census 2000. Gazetted areas with a combined population of 10,000 or more were defined as urban areas; while areas with a population of less than 10,000 were classified as rural [27]. Both urban and rural communities were selected with the aim of achieving within-community homogeneity in demographic and socioeconomic profiles. In view of the fact that this study was designed as a prospective cohort study, these communities were also selected based on the pragmatic requirement of optimizing the capacity of investigators to maintain long-term follow-up of participants and cooperation by community leaders. These factors were important to ensure the continuity of data collection, which is scheduled every 3 years, for a period of 15 years.

### 2.3. Subject Recruitment

The participants were adults between the ages of 18 to 90 years old. The sampling process involved a four-stage sampling process; first, the states were selected; second, selection of the ‘communities’; third followed by households within the community and fourth, the individuals’ selection within the households. The seven states were chosen to ensure adequate representation of the major ethnic groups in Malaysia. The methods of recruitment were standardized. Participants were recruited by announcements and written invitations through local community leaders. All household members aged ≥18 years old were invited to attend screening sessions at the chosen local community centers. More than 20,000 invitations were sent out to all the selected states’ communities with a response rate of 60–70% recorded at each site. The response rate was considered a good outcome, as the community had been approached and informed earlier about the screening program, and the process was facilitated by each of the community representatives; namely, their community committee or head of the villagers. On the day of the screening program, participants were given an information leaflet about the program, and individuals were screened for eligibility. Informed consent was obtained from those who were eligible and willing to participate. Participants were given information regarding the purpose of the study and were requested to fast for at least 8 h prior to the screening.

### 2.4. Data Collection and Study Procedures

The baseline data was collected from 2007 to 2017. The cross-sectional analytic sample presented in this paper consisted of 13,654 participants who were recruited at baseline. Out of the 13,654 participants, 7479 had data on the physical activity components and were therefore included in the data analysis.

#### 2.4.1. Data Collection Process

All researchers, interviewers, and investigators were trained. The data collection process and procedures were standardized prior to the conduct of the study, to ensure minimal variability during data collection and its outcome. Standardized, pre-tested, interviewer-based case report forms and relevant questionnaires were used to collect information regarding age, gender, ethnic group, occupation, educational attainment, marital status and smoking status. Participants were required to report on their underlying medical history, and current treatment; particularly non-communicable diseases such as hypertension, dyslipidaemia, type 2 diabetes, heart disease or ischemic heart disease and stroke.

#### 2.4.2. Study Tools and IPAQ

The questionnaire used for the assessment of physical activity was the Long International Physical Activity Questionnaire (Long-IPAQ) in English and Malay version. The measure of physical activity for clinical or research use has been a challenge, and although there is no international consensus on the correct method of describing physical activity levels, an attempt was made by the international community in 1998, Geneva, to develop an international measure for physical activity, which led to the development of the International Physical Activity Questionnaire (IPAQ) in 2000 [28]. The IPAQ has since been translated into various languages and used as a standardized measurement of physical activity all over the world. The long IPAQ has been translated into the Malay language and is freely available: https://sites.google.com/site/theipaq/questionnaire_links (accessed on 13 November 2022) [29].

Physical activity level is classified according to the International Physical Activity Questionnaire (IPAQ)-Long form as low, moderate and high. The test–retest reliability and validity of IPAQ-M was good for the evaluation of physical activity. The intraclass correlation co-efficient (ICC) scores showed moderate to good correlations (ICC = 0.54–0.92; *p* < 0.001) on items categorized by intensities and domains and a Kappa (κ) of 0.73 for total activity. The validity results from the PA-Log were statistically significant (*p* < 0.001) across intensities and domains (ρ = 0.67–0.98)) (ρ = Spearman’s correlation coefficient) [30].

The IPAQ is a self-administered questionnaire measuring the subject’s 1-week total physical activity. The total physical activity is calculated as a total of four domains, which are work, active transportation, domestic and yard work, and leisure time related. The total work domain is calculated as the sum of walking, and moderate and vigorous activity performed during work; the total active transport domain is the sum of walking and cycling undertaken as means for transportation; the domestic and yard work domain is the sum of vigorous and moderate yard work, and moderate domestic (indoor chores) work; and lastly, the leisure time domain is the sum of walking, moderate and vigorous activity perform during leisure time. These are reported in metabolic equivalents (MET) × minutes per week. In the long IPAQ, each domain activities are specified and the days/hours/minutes are calculated. The total physical activity was categorized as low (<600 MET × minutes per week), moderate (600–3000 MET × minutes per week), and high (>3000 MET × minutes per week) physical activity, corresponding to insufficiently active, sufficiently active and very active, respectively, according to the PA guideline recommended by WHO [31].

#### 2.4.3. Anthropometry and Clinical Measurements

Anthropometric measures including weight (kg), height (cm), body mass index (BMI) calculation, waist circumference (WC), waist–hip ratio (WHR) and blood pressure (BP) were obtained as described in previous publications [23,24,25,26]. Weight and height were measured using standardized weight and height scales (Seca, Germany). Subjects were asked to remove all heavy clothing and remain bare footed with arms hanging freely by their sides. Weight was recorded in kilograms (kg) and height in centimeters (cm) to the nearest decimal point. WC and WHR were measured to the nearest 0.1 cm by using non-stretchable measuring tape with the subjects standing in a relaxed position and arms at the side. The measurement was taken at the midpoint between the lower rib margin (12th rib) and the iliac crest. For hip circumference, the measurement is taken around the pelvis at the point of maximal protrusion of the buttocks.

The blood pressure was measured on two occasions at five minutes apart on the right arm supported at heart level, while participants were seated using an Omron automatic digital blood pressure monitor (Omron HEM-757). Participants were advised not to smoke, exercise or eat in the last 30 min, not to climb stairs in the last 15–30 min, and were made to rest for at least five minutes before the measurements were taken. Right arm BP measurements were taken two minutes apart, and a mean of the two readings was taken as the BP for the participant. The average of the two BP readings was used for analysis. If the measurements differ by 5 mmHg of either systolic or diastolic readings, subsequent measurements were taken at 5–10 min apart. The process was repeated until two BP values, which did not differ by more than 5 mmHg of either systolic or diastolic readings, were obtained. The average of these two BP readings was used as the BP value for that particular subject.

#### 2.4.4. Other Socio-Demographic Factors

Other socio demographic definitions are education attainment and occupation. For education levels, this was categorized as ‘no formal education’, ‘primary’, ‘secondary’ and ‘tertiary’. Participants who had never been to school to get any form of education were categorized into ‘no formal education’, while ‘primary’ education level represented those who had completed standard one to six years of schooling or age between 7 to 12 years old during schooling at primary school. ‘Secondary’ education level represented those who completed form one to lower or upper six form or age between 13 to 19 years old during schooling at secondary school. The ‘tertiary’ education level represented those who attended colleges or universities or attained post-schooling certificates, diplomas, degrees or higher.

Occupations were categorized based on the Malaysian Standard Classification of Occupations (MASCO) 2020 groups; group 1 are legislators, business and senior office managers; group 2 are professionals, teaching and health; group 3 are technicians; group 4 are clerks; group 5 are service workers, shops, market and sales; group 6 are skilled agricultural; group 7 are crafts-related trade workers; group 8 are plant machine operators; group 9 are elementary occupation laborers; group 10 are armed forces; and lastly group 11 are homemaker, housewife and house husband [32]. These categories are then re-categorized according to their energy expenditure in relevance to their occupation: Professional, Technical, Clerical, Elementary and Housewife/unemployed/student.

Income was categorized according to the Malaysia Household Income and Basic Amenities Survey Report 2016 from the Department of Statistics Malaysia. The household groups that exist in the income distribution structure house in Malaysia were classified into three categories: the bottom 40% (B40), medium 40% (M40) and top 20% (T20). B40 households are households with an income below RM4360, M40 households are households with an income between RM4360–RM9619 and the T20 home group has an income exceeding RM9619 [33].

#### 2.4.5. Clinical Characteristics

Current smokers were defined as participants who were currently smoking cigarettes or had smoked cigarettes within the past five years. Ex-smokers were those who had stopped smoking for more than five years, and non-smokers were those who had never smoked.

BMI was categorized according to the Malaysia Clinical Practice Guideline on the Management of Obesity, 2004. Underweight was defined as BMI < 18.5 kg/m^2^, normal BMI, range as BMI of 18.5–22.9 kg/m^2^, overweight as BMI of ≥23–27.4 kg/m^2^ and obesity as BMI ≥ 27.5 kg/m^2^ [34].

For abdominal obesity, waist circumference (WC) categories were based from International Diabetes Federation with normal WC is defined as <90 cm for men and <80 cm for women, and abnormal WC is defined as ≥90 cm for men ≥80 cm for women [35]. The waist hip ratio (WHR), on the other hand, is categorized as follows; normal WHR is defined as <0.9 for men and <0.8 for women, and abnormal WHR is defined as men WHR ≥ 0.9 and women WHR ≥ 0.8 [36].

Hypertension was defined according to the Malaysia Clinical Practice Guideline on the Management of Hypertension, 5th Edition, 2018 [37]. Hypertension was defined if there is the presence of either; i. average systolic BP ≥ 140 mmHg and/or average diastolic BP ≥ 90 mmHg; ii. the participants reported a history of hypertension; or iii. participants reported taking anti-hypertensive medications.

Diabetes was defined according to the Malaysia Clinical Practice Guideline on the Management of Type 2 Diabetes Mellitus 5th Edition 2015. Diabetes was defined if there is the presence of either; i. fasting plasma glucose at ≥7.0 mmol/L and/or self-reported diabetes, and/or taking medications for diabetes [38].

Dyslipidaemia was defined according to the Malaysia Clinical Practice Guideline on the Management of Dyslipidaemia 5th Edition, 2017 [39]. Dyslipidaemia was defined if any abnormalities based on the following cut-off points; i. total cholesterol >5.2 mmol/L, LDL > 3.4 mmol/L, HDL < 1.0 mmol/L (men), HDL < 1.2 mmol/L (women), TG > 1.7 mmol/L OR ii. on treatment for dyslipidaemia. The methods of serum lipid profile analysis (total cholesterol (TC), triglycerides (TG), high-density lipoprotein cholesterol (HDL-c), low-density lipoprotein cholesterol (LDL-c)) was by using an automated clinical chemical analyzer (Cobas Integra 400 plus, Roche Diagnostic, Basel, Switzerland). LDL-c was calculated using the Freidewald equation (for TG ≤ 4.5 mmol/L). Non-HDL-c was calculated using the following equation: TC—HDL-c (mmol/L). Although this guideline categorized elevated LDL-c according to cardiovascular risk disease (CVD) risk profiles; if LDL-c > 1.8 mmol/L is for the very high-risk category, >2.6 mmol/L for high-risk category, >3.4 mmol/L for intermediate risk group and low-risk category; the guidelines specify that the LDL-c cut-off point is >3.0 mmol/L. The recommended lipid therapy is based on joint decision making between prescribers and patients; taking into account the risk and benefits. However, for the purpose of this study, the highest level, i.e., LDL-c > 3.4 mmol/L, was chosen to define “elevated LDL-c”; as above this threshold, lipid therapy is indicated for patients from intermediate, high risk, and very-high risk categories.

### 2.5. Statistical Analysis

Data were analyzed using SPSS software version 24. Numerical variables were described using mean (±Standard Deviation [SD]). Categorical variables were described with frequency and percentage. Significance level was set at *p* < 0.05 with a 95% Confidence Interval. The physical activity level was calculated according to the IPAQ MET-min per week and described using median, as per recommended by the IPAQ guideline. Total physical activity was categorized according to insufficiently active (low), sufficiently active (moderate) and very active (high) physical activity. Multinomial regression was used to determine the association between sociodemographic, clinical factors and level of physical activity.

### 2.6. Ethical Approval

Ethical approval of the study protocol was obtained from the institutional Research Ethics Committee (REC) of Universiti Teknologi MARA (UiTM) Selangor, Malaysia and the Malaysia Research Ethics Committee (MREC) from the National Medical Research Register (NMRR). The approval reference number from UiTM REC is [REC/UITM/2007] and NMRR Project ID: NMRR-11-679-8846.

## 3. Results

A total of 13,654 participants were involved in the study. From this sample, 7479 participants had completed the International Physical Activity Questionnaire (IPAQ) long form and were therefore included in the data analysis.

Table 1 shows the sociodemographic and clinical characteristics of the participants within the physical activity levels. The mean age was 51.68 (±9.5 SD) with the majority in the middle age group (40–60 years old). Most participants are Malay (76.9%), female (56.9%), married (90.5%), rural dwellers (60.15%) in the B40 income category (88.1%) and have received at least a primary education level (39.2%). In terms of clinical characteristics, the majority were in the overweight and obese category (74.1%), have hypertension (52.6%), and have abnormal total cholesterol (64.5%) and LDL (57.1%) levels. This is also reflected in all three of the physical activity levels.

Table 2 presents the physical activity in MET × minutes per week. The total reported physical activity in median (IQR) was 1584.0 (0–5637.3) MET-min per week, with median for insufficiently active PA was 0 (0–0) MET-min per week, sufficiently active PA was 1485.0 (947.8–2190.0) MET-min per week and very active PA was 7854.0 (4851.0–14,746.5) MET-min per week. Comparing between the four domains, the highest total for PA was in the domestic domain, which is 490 (0–2400) MET-min per week, followed by transport (0 (0–396) MET-min per week), leisure (0 (0–320) MET-min per week) and working domain (0 (0–0)).

Table 3 presents the factors associated with sufficiently active and very active PA levels. Participants from the urban location were 1.7 and 1.6 times more likely to engage in sufficiently active and very active PA, respectively, compared to the participants in the rural location. Malay ethnicity are 1.6 and 1.5 times more likely to perform sufficiently active and very active PA, respectively, compared to the Indigenous ethnic group. Those with no formal education are 1.4 times more likely to participate in sufficiently active PA compared to those with tertiary education. Participants in the elementary occupation group are 1.3 times more likely to have very active PA levels compared to the housewife/unemployed/student group. Current smokers are 1.3 and 1.5 times more likely to participate in sufficiently active and very active PA, respectively, compared to non-smokers. Lastly, those with high HDL are 1.2 times likely to perform sufficiently active and very active PA compared to those with low HDL.

Those in the low-income (B40) category are 46% less likely to engage in sufficiently active PA compared to those in the higher income (T20) category. Males are 35% less likely to participate in sufficiently active PA and 28% less likely to participate in very active PA compared to females. It is also noted that participants with normal BMI are 27% less likely to engage with very active PA compared to those who are obese.

Other clinical factors such as waist circumference, blood pressure and total cholesterol were only found to be significant in univariate analysis. However, when included in the final model using multivariate analysis, they are not found to be significant factors for sufficiently active and very active PA.

Triglyceride level, LDL levels, diabetes status, history of stroke and IHD were not presented, as these factors are not significant during univariate analysis. Thus, we have only included the significant factors from univariate and multivariate in Table 3.

## 4. Discussion

It is estimated that about 27.5% of the world’s population is physically inactive [12]. Our study findings concur that Malaysians have a high prevalence of insufficiently active PA (37.3%). This prevalence is higher in comparison with the 2019 National Health and Morbidity Survey (NHMS), which reported that 25.1% of Malaysians are physically inactive [40]. In contrast, well-developed countries such USA, Canada, Australia and New Zealand have recorded higher levels of PA, with more than 50% of their population performing very active PA [41]. This high prevalence of insufficiently active PA levels from our findings is influenced by multifactorial components associated with PA [18].

The physical activity levels according to the four IPAQ domains showed that the highest PA was documented in the domestic domain. This is similar to a Malaysian study by Chu HY and Moy FM (2014), which demonstrates that the participants are most active in the domestic physical activity domain, followed by occupation, transportation and leisure [21]. This is in contrast with studies from Hungary and Ghana; whereby the highest PA is found in the working domain [42,43]. This similar pattern is also noted in lower income countries, with more activities undertaken at work and for transport [44]. However, in our study, the work, leisure and transport domain had recorded lower METs. The low PA levels in the transport domain could be related to the excess use of motorized transportation such as cars and motorcycles [20]. As for the leisure domain, the reason for this may be due to lack of facilities for PA, concern for safety, especially amongst women, and lack of time to fit in additional PA on top of other domestic and occupational commitments [45,46,47,48,49]. To counter this, guidelines and policies are in place to encourage Malaysians to be more active; these include the National Strategic Plan for Active Living [16,50], improving facilities such as recreational parks and sport facilities, and accessibility for non-motorized transportation such as covered walkways and cycling zones [45,51]. A study conducted in China showed that with an increase of park facilities usage, there is an improvement in leisure time PA [52].

The result from our study demonstrated that participants from the urban location are more likely to engage in sufficiently active and very active PA compared to those in the rural location. A study in an urban location [53] and another study in a rural location also had similar findings [43]. Physical activity is a complex behavior that is associated with personal preference, social and environmental factors [41,54]. When these factors were examined, it is found that the place to exercise is the most crucial [55]. It is identified that among urban populations; access to parks, specific walking trails, and exercise equipment was favorable for sufficiently active and very active PA [55]. The reduction in PA among the rural dwellers is found to be related to the spread of technology and the shift of lifestyle; for example, from agriculture-based activities to industries and services [56]. To improve the PA levels among rural communities, new project developments that incorporate PA areas such as parks with exercise facilities will improve outcome [57].

There is an association between Malay ethnicity with both sufficiently active and very active PA levels. Malays are more likely to engage in sufficiently active and very active PA compared to the indigenous groups. This is in contrast with a previous study by Teh CH et al., 2014, where the indigenous groups were found to be more physically active compared to the other ethnic groups, including Malay [58]. This apparent disparity could be due to modernization in terms of lifestyle and behavior, with most people spending more leisure time at home and having less vigorous work compared to before. The upgrade in infrastructure nearer to their communities also made them less mobile, decreasing their need to travel more [59]. Environmental factors also play a barrier in performing physical activity such as poor maintenance of natural resources, lack of access to sport facilities and suitable walking area [60]. This is not only the trend among the indigenous in Malaysia, but also worldwide [61,62].

Our studies also found that those with lower education levels are more physically active. In another Malaysian study, it was found that higher education level did not correlate with higher physical activity, and that educated Malaysians do not see physical activity as an important aspect of life or leisure [18]. Lower education level is also related to skill levels that are matched with the elementary occupation category, which mainly involves manual labor [32]. Our study also concurs with this, as those in the elementary occupation category are more likely to have higher PA. Previous studies have shown that individuals with a physically demanding occupation that requires manual labor reported higher total physical activity levels compared to those with higher status jobs that do less manual labor [63,64]. A more detailed correlation is needed to assess those with low education levels and their occupation, to determine whether that influences their PA.

Vast research that was done in high income countries showed that socioeconomic status is an important factor to PA [65]. This concur with our finding, which showed that those earning low income were less likely to engage in adequate PA. A possible explanation may be because those who earned a higher income have better access to exercise facilities, such as membership to the gymnasium [65]. Higher income can create opportunities for them to buy their own health equipment, which contributes to more PA [57,66]. Better opportunities are needed for the housing areas of the low-income population to have their own exercise facilities that are safe and freely accessible. Hua, Ying et al. found that there is a necessity for a better sustainable building to support healthy lifestyle and wellbeing [67]. Most of the low-income population may spend more time in their workplace and do not have the time to increase PA; therefore, creating opportunities to exercise within their workplace areas is crucial [68]. A study done by Bergman, Frida et al. in 2018 showed that there is an increase in walking time in workplaces when there is a treadmill available onsite [69].

This study found that smokers are more likely to perform sufficiently active and very active PA compared to non-smokers. This is contrary to the findings from a systematic review that smokers have low PA [70]. A more recent literature overview also concluded that there is an inverse relationship between exercise and smoking behaviors [71]. This discrepancy may be explained by the fact that smokers are from the younger age group and thus, more physically active [72]. However, this is beyond the scope of our study.

Our findings also noted that men are less likely to participate in sufficiently active and very active PA compared to women. This is in contrast with previous studies, which showed females were less active compared to males [18,22,58,73]. The reason for this could be because more women compared to men were involved in domestic-related PA, which accounts for the biggest PA domain in this population. This is supported by a previous study, which showed that only 20.4% would achieve the recommended PA level compared to 42.7% if domestic PA is excluded from the total MVPA [74]. Another study using both activity recall and accelerometers also documented that the activities that generated a significant proportion of bouts of MVPA revolved around housework and childcare at home [46]. A study comparing exercise habits and total levels of energy expenditure found that women reported more METs from moderate intensity activity compared to men, with the reason for exercise mainly being for weight-related reasons, toning their bodies and for health [75].

Our participants with normal BMI are less likely to engage in very active PA compared to those who are obese. This is similar to a previous health survey, which showed that among women, the overweight/obese group reported a slightly higher prevalence of high PA compared to the normal-weight group. However, the opposite is true for men, which reported higher PA among the normal weight group [76]. Another study found that although healthy obese adults had higher total PA compared to unhealthy obese adults; they were less likely to meet the recommended MVPA compared to healthy normal-weight adults [77]. The contrasting findings in our study could be due to several reasons; those who are obese and overweight are more driven to perform more PA as part of weight management, physical/functional fitness and social support [48,78], while those with normal BMI may perceive their normal BMI as proof of adequate PA [79] and therefore are less likely to engage in high PA.

In terms of lipid profile, our study found that participants with high HDL are more likely to perform sufficiently active and very active PA. This could reflect the results of performing regular moderate and high PA as many studies have identified that high PA is associated with increase in HDL levels [80]. In fact, some studies suggest that a short bout of exercise < 10 min can increase HDL level [81].

## 5. Strength and Limitations

The strength of this study is the large sample size and participants from various localities that provided good representation of the population. Recruitment is via voluntary participation. This study uses the structured IPAQ to measure physical activity level and its distribution across different domains, which is useful in capturing holistic view in terms of PA. The limitation of this study is that it uses a self-reported questionnaire among the participants and this may lead to recall bias. The methodology of this study is cross-sectional and therefore, it only reflects the association between participants characteristic and not the causal relationship. The results should be interpreted in this context. The follow-up data of this ongoing prospective cohort study would be able to offer more findings in the future.

## 6. Conclusions

This study concludes that the PA level in Malaysia is still insufficient. The factors that are associated with sufficiently active and very active PA are living in urban area, having no formal education, elementary occupation, current smokers and having high HDL. Whereas rural area, low income, male and normal BMI are less likely to participate in sufficiently active and very active PA. Therefore, there is a need to promote PA among individuals in the rural area, low income, male and normal BMI. At the individual level, encouragement by giving health promotion and personalized consultation can be employed by health professionals. However, there is also a need to involve the workplace and policymakers to support and provide PA-friendly infrastructures and facilities. This can include parks with running and cycling paths, exercise facilities and equipment, and to encourage non-motorized transportation, walking and work-based physical activity.

## Figures and Tables

**Table 1:** The socio-demographic and clinical characteristics of the participants within the physical activity levels (insufficiently active, sufficiently active, very active).

Demographic Characteristics Categories	Total Sample (%)	Insufficiently Active (*n* = 2793)	Sufficiently Active (*n* = 1954)	Very Active (*n* = 2612)	*p* Value
All subjects, *n* (%)	7479 (100%)	2793 (37.3%)	1937 (25.9%)	2749 (36.8%)	0.001 *
Mean age in years (±SD)	51.68 (±SD 9.5)	52.73 ±(SD9.7)	51.58 (±SD 9.3)	50.68 (±SD 9.3)	0.001 *
Age group (years), *n* (%)					
<30	32 (0.4)	9 (0.3)	5 (0.3)	18 (0.7)	0.001 *
30–<40	728 (9.7)	248 (8.9)	182 (9.4)	298 (10.8)	
40–<50	2490 (33.3)	845 (30.3)	667 (34.5)	978 (35.6)	
50–<60	2516 (33.6)	934 (33.5)	647 (33.4)	935 (34.0)	
≥60	1711 (22.9)	756 (27.1)	435 (22.5)	520 (18.9)	
Gender, *n* (%)					
Male	3226 (43.1)	1232 (44.1)	751 (38.8)	1243 (45.2)	0.001 *
Female	4252 (56.9)	1561 (55.9)	1185 (61.2)	1506 (54.8)	
Marital status, *n* (%)					
Single	145 (2.0)	42 (1.5)	49 (2.6)	54 (2)	0.003 *
Married	6642 (90.5)	2461 (90.3)	1694 (89)	2487 (91.7)	
Divorcee/widowed/separated	552 (7.5)	221 (8.1)	161 (8.5)	170 (6.3)	
Location, *n* (%)					
Urban	2988 (39.9)	822 (29.4)	960 (49.6)	1206 (43.9)	0.001 *
Rural	4491 (60.1)	1971 (70.6)	977 (50.4)	1543 (56.1)	
Income, *n* (%) ^Ψ^ (*n* = 6052)					
B40	5571 (88.1)	2165 (93.7)	1369 (83.2)	2037 (86.1)	0.001 *
M40	641 (10.1)	124 (5.4)	228(13.9)	289 (12.2)	
T20	110 (1.7)	21 (0.9)	49 (3.0)	40 (1.7)	
Ethnic Group, *n* (%)					
Malay	5747(76.9)	1976 (70.7)	1586 (82)	2185 (79.5)	0.001 *
Chinese	270 (3.6)	82 (2.9)	84 (4.3)	104 (3.8)	
Indian	103 (1.4)	28 (1.0)	34 (1.8)	41 (1.5)	
Others (Indigenous)	1355(18.1)	707 (25.3)	231 (11.3)	417 (15.2)	
Educational level, *n* (%) ^Ψ^ *n* = 7026					
No formal education	1127 (14.4)	256 (9.4)	384 (20.2)	487 (18)	0.001 *
Primary	2868 (39.2)	955 (35.2)	786 (41.4)	1127 (41.6)	
Secondary	2260 (30.9)	973 (35.9)	499 (26.3)	788 (29.1)	
Tertiary	1065 (14.5)	528 (19.5)	228 (12)	309 (11.4)	
Occupation, *n* (%)					
Professional	492 (6.9)	110 (4.2)	177 (9.5)	205 (7.7)	0.001 *
Technical	967 (13.5)	302 (11.5)	279 (14.9)	386 (14.5)	
Clerical	394 (5.5)	113 (4.3)	130 (7.0)	151 (5.7)	
Elementary	2433 (34.1)	958 (36.6)	522 (27.9)	953 (35.9)	
Housewife/unemployed/student	2855 (40)	1133 (43.3)	760 (40.7)	962 (36.2)	
Smoking, *n* (%)					
Never	5589 (75.5)	2090 (75.7)	1505 (78.8)	1994 (73.1)	0.001 *
Previous	798 (10.8)	310 (11.2)	188 (9.8)	300 (11)	
Current	1013 (13.7)	361 (13.1)	218 (11.4)	434 (15.9)	
Waist Circumference, *n* ^Ψ^ (%) (*n* = 7026)					
Normal	3173 (43.5)	1210 (44.8)	773 (40.3)	1190 (44.3)	0.05
Abnormal	4125 (56.5)	1488 (55.2)	1143 (59.7)	1494 (55.6)	
Waist Hip Ratio, *n* (%)					
Normal	2631 (35.9)	917 (33.8)	691 (36.3)	1023 (37.8)	0.009 *
Abnormal	4690 (64.1)	1793 (66.2)	1215 (63.7)	1682 (62.2)	
BMI, *n* (%) ^Ψ^ *n* = 7049					
Underweight (<18.5 kg/m^2^)	242 (3.3)	113 (4.2)	51 (2.7)	78 (2.9)	0.001 *
Normal (18.5–<23 kg/m^2^)	1664 (22.6)	683 (25.1)	399 (20.8)	582 (21.4)	
Overweight (23–<27.5 kg/m^2^)	2809 (38.2)	994 (36.5)	767 (40)	1048 (38.6)	
Obese (>27.5 kg/m^2^)	2639 (35.9)	932 (34.2)	702 (36.6)	1005(37)	
Diabetes status, *n* (%)					
Non-diabetic	5905 (82.6)	2162 (81.8)	1542 (82.8)	2201 (83.4)	0.311
Diabetic	1240 (17.4)	481 (18.2)	320 (17.2)	439 (16.6)	
Dyslipidaemia, *n* (%)					
No	1166 (15.7)	452 (16.3)	278 (14.4)	436 (16)	0.211
Yes	6269 (84.3)	2327 (83.7)	1647 (85.6)	2295 (84)	
Total cholesterol, *n* (%)					
Normal	2581 (35.5)	1010 (37.3)	626 (33.1)	945 (35.4)	0.013 *
Abnormal	4691 (64.5)	1698 (62.7)	1266 (66.9)	1727 (64.6)	
HDL level, *n* (%)					
Low HDL	2833 (39)	1151 (42.5)	687 (36.3)	995 (37.2)	0.001 *
High HDL	4439 (61)	1557 (57.5)	1205 (63.7)	1677 (62.7)	
Triglyceride level, *n* (%)					
Normal	4093 (56.3)	1486 (54.9)	1076 (56.9)	1531 (57.3)	0.171
Abnormal	3177 (43.7)	1221 (45.1)	815 (43.1)	1141 (42.7)	
LDL level, *n* (%)					
Not elevated	3114 (42.9)	1200 (44.3)	784 (41.5)	1130 (42.4)	0.135
Elevated	4147 (57.1)	1507 (55.7)	1104 (58.5)	1536 (57.6)	
Blood pressure, *n* (%)					
No hypertension	3494 (47.4)	1234 (45.1)	907 (47.3)	1353 (49.7)	0.003 *
Hypertension	3880 (52.6)	1500 (54.9)	1010 (52.7)	1370 (50.3)	
History of stroke, *n* (%)					
yes	96 (1.3)	44 (1.6)	24 (1.3)	28 (1.0)	0.175
no	7303 (98.7)	2716 (98.4)	1887 (98.7)	2700 (99)	
History of IHD, *n* (%)					
yes	344 (4.7)	142 (5.1)	85 (4.4)	117 (4.3)	0.288
no	7056 (95.3)	2619 (94.9)	1827 (95.6)	2610 (95.7)	

Statistical analysis: Chi Square test. * Statistically significant at α = 0.05 level. ^Ψ^ Number do not equal to *n* due to missing data.

**Table 2:** Distribution of insufficiently active, sufficiently active and very active physical activity across total physical activity and its 4 domains based on IPAQ.

Physical Activity	Median	IQR
Total Physical activity MET-Min/week	1584.00	0–5637.25
Insufficiently active PA (n = 2793)	0	0–0
Sufficiently active PA (n = 1937)	1485.00	947.75–2190.00
Very active PA (n = 2749)	7854.00	4851.00–14,746.50
Working domain (WD) MET-min/week	0	0–0
Transport domain (TD) MET-min/week	0	0–396
Domestic domain (DD) MET-min/week	490.00	0–2400.00
Leisure domain (LD) MET-min/week	0	0–320.00
Total WalkMET	148.50	0–891
Total ModMET	770.00	0–3258.00
Total VigMET	0	0–0

Data are median (IQR). MET = metabolic equivalents. Insufficiently active (Low) physical activity <600 MET × min per week. Sufficiently active (Moderate) physical activity 600–3000 MET × min per week. Very active (High) physical activity >3000 MET × min per week.

**Table 3:** Factors associated with PA categories (Insufficiently active/Sufficiently active/Very active) (reference category Insufficiently active PA).

Variable	Univariate Analysis				Multinomial Analysis			
	Sufficiently Active PA		Very Active PA		Sufficiently Active PA		Very Active PA	
	Crude OR ^a^ (95% CI)	*p* Value	Crude OR ^a^ (95% CI)	*p* Value	Crude OR ^b^ (95% CI)	*p* Value	Crude OR ^b^ (95% CI)	*p* Value
Age	0.987 (0.981, 0.993)	<0.001	0.977 (0.972, 0.983)	<0.001	-	-	-	-
Age group								
<30	0.966 (0.322, 2.899)	0.950	1.275 (1.019, 1.597)	0.034	-	-	-	-
30–<40	1.372 (1.174, 1.603)	<0.001	1.204 (1.031, 1.405)	0.019	-	-	-	-
40–<50	2.908 (1.296, 6.523)	0.010	1.747 (1.427, 2.138)	<0.001	-	-	-	-
50–<60	1.683 (1.456, 1.945)	<0.001	1.455 (1.260, 1.681)	<0.001	-	-	-	-
≥60	1							
Gender								
Male	0.803 (0.714, 0.904)	<0.001	1.046 (0.941, 1.163)	0.408	0.649 (0.534, 0.791)	<0.001	0.721 (0.603, 0.862)	<0.001
Female	1							
Marital status								
Single	1.601 (1.012, 2.535)	0.045	1.671 (1.666, 2.622)	0.025	-	-	-	-
Married	0.945 (0.764, 1.168)	0.601	1.314 (1.067, 1.617)	0.010	-	-	-	-
Divorcee/ widowed/ separated	1							
Location								
Urban	2.356 (2.088, 2.658)	<0.001	1.874 (1.677, 2.094)	<0.001	1.658 (1.401, 1.962)	<0.001	1.583 (1.355, 1.849)	<0.001
Rural	1							
Income								
B40	0.271 (0.162, 0.454)	<0.001	0.494 (0.290, 0.841)	0.009	0.541 (0.295, 0.991)	0.047	-	-
M40	0.788 (0.452, 1.374)	0.401	1.224 (0.693, 2.160)	0.487	0.883 (0.486, 1.604)	0.683	-	-
T20	1							
Ethnic Group								
Malay	2.457 (2.088, 2.890)	<0.001	1.875 (1.637, 2.147)	<0.001	1.628 (1.313, 2.020)	<0.001	1.454 (1.207, 1.751)	<0.001
Chinese	3.135 (2.235, 4.399)	<0.001	2.150 (1.571, 2.943)	<0.001	1.378 (0.870, 2.182)	0.172	1.304 (0.853, 1.993)	0.220
Indian	3.716 (2.206, 6.262)	<0.001	2.483 (1.513, 4.075)	<0.001	1.452 (0.781, 2.699)	0.238	1.281 (0.709, 2.314)	0.411
Others (Indigenous)	1							
Educational level								
No formal education	3.474 (2.783, 4.336)	<0.001	3.251 (2.644, 3.996)	<0.001	1.443 (1.003, 2.076)	0.048	-	-
Primary	1.906 (1.589, 2.286)	<0.001	2.016 (1.710, 2.378)	<0.001	1.250 (0.963, 1.622)	0.093	-	-
Secondary	1.188 (0.983, 1.435)	0.075	1.384 (1.169, 1.638)	<0.001	0.997 (0.784, 1.268)	0.979	-	-
Tertiary	1							
Occupation								
Professional	2.399 (1.859, 3.096)	<0.001	2.195 (1.714, 2.810)	<0.001	-	-	1.067 (0.748, 1.524)	0.719
Technical	1.377 (1.142, 1.660)	0.001	1.505 (1.266, 1.790)	<0.001	-	-	1.147 (0.905, 1.453)	0.258
Clerical	1.715 (1.311, 2.243)	<0.001	1.574 (1.215, 2.038)	0.001	-	--	1.110 (0.828, 1.488)	0.485
Elementary	0.812 (0.706, 0.935)	0.004	1.172 (1.035, 1.327)	0.012	-	-	1.330 (1.111, 1.591)	0.002
Housewife/unemployed/student	1							
Smoking								
Never	1		-		1		1	
Previous	0.842 (0.694, 1.021)	0.081	1.014 (0.856, 1.203)	0.870	1.128 (0.891, 1.428)	0.315	1.145 (0.927, 1.413)	0.210
Current	0.839 (0.700, 1.005)	0.056	1.260 (1.082, 1.468)	0.003	1.264 (1.005, 1.589)	0.045	1.482 (1.216, 1.807)	<0.001
Waist Circumference								
Normal	0.832 (0.739, 0.936)	0.002	0.980 (0.880, 1.091)	0.706	-	-	-	-
Abnormal	1		1		-	-	-	-
Waist Hip Ratio								
Normal	1.112 (0.984, 1.257)	0.090	1.189 (1.064, 1.329)	0.002	1.184 (0.996, 1.406)	0.055	-	-
Abnormal	1		1		1		-	-
BMI								
Underweight (<18.5 kg/m^2^)	0.599 (0.424, 0.846)	0.004	0.640 (0.473, 0.866)	0.004	-	-	-	-
Normal (18.5–<23 kg/m^2^)	0.776 (0.662, 0.908)	0.002	0.790 (0.686, 0.911)	0.001	-	-	-	-
Overweight (23–<27.5 kg/m^2^)	1.024 (0.894, 1.174)	0.728	0.978 (0.863, 1.107)	0.723	-	-	0.945 (0.803, 1.112)	0.497
Obese (>27.5 kg/m^2^)	1		1		-	-	1	
Total cholesterol								
Normal	0.831 (0.735, 0.941)	0.003	0.920 (0.823, 1.028)	0.141	-	-	-	-
Abnormal	1		-		-	-	-	-
HDL level								
Low HDL	1				1		-	
High HDL	1.297 (1.149, 1.463)	<0.001	1.246 (1.117, 1.390)	<0.001	1.217 (1.050, 1.412)	0.009	1.152 (1.008, 1.318)	0.038
Blood pressure								
No hypertension	1				-		-	
Hypertension	1.092 (0.971, 1.227)	0.142	1.200 (1.079, 1.335)	0.001	-		-	

^a^ Statistical test: Simple logistic regression. ^b^ Statistical test: Multinomial logistic. *p* value is significant at the 0.05 level (2-tailed).

## Data Availability

The data and samples can be assessed by permission through the principal investigator, Dato’ Khalid Yusoff (2008–2021), Nafiza Mat Nasir (2021–current), Faculty of Medicine, Universiti Teknologi MARA, Sungai Buloh 47000, Selangor, Malaysia. Data is kept in the data room, Centre for Translational Research and Epidemiology (CenTRE), Level 2, Clinical Training Block, Faculty of Medicine, Universiti Teknologi MARA, Sungai Buloh, Jalan Hospital, Selangor 47000, Malaysia.
